# Astaxanthin Inhibits Proliferation and Induces Apoptosis of Human Hepatocellular Carcinoma Cells via Inhibition of Nf-Κb P65 and Wnt/Β-Catenin *in Vitro*

**DOI:** 10.3390/md13106064

**Published:** 2015-09-24

**Authors:** Jingjing Li, Weiqi Dai, Yujing Xia, Kan Chen, Sainan Li, Tong Liu, Rong Zhang, Jianrong Wang, Wenxia Lu, Yuqing Zhou, Qin Yin, Huerxidan Abudumijiti, Rongxia Chen, Yuanyuan Zheng, Fan Wang, Jie Lu, Yingqun Zhou, Chuanyong Guo

**Affiliations:** 1 Department of Gastroenterology, Shanghai Tenth People’s Hospital, Tongji University School of Medicine, Shanghai 200072, China; E-Mails: sealjj@126.com (J.L.); dai_yue@163.com (W.D.); gagaxyj@126.com (Y.X.); cutking@126.com (K.C.); Lrk678@126.com (S.L.); klmn1334@sina.com (T.L.); Sylvia_rong@163.com (R.Z.); hellowangjr@163.com (J.W.); 15214327248@163.com (W.L.); zyq937065339@163.com (Y.Z.); yinqin201011@163.com (Q.Y.); hurxida0101@126.com (H.A.); tjchenrongxia@hotmail.com (R.C.); sxzhengyuanyuan@126.com (Y.Z.); fairywong04285@163.com (F.W.); kennisren@hotmai.com (J.L.); 2 The First Clinical Medical College of Nanjing Medical University, Nanjing 210029, China; 3 The First Affiliated Hospital of Soochow University, Suzhou 215006, China

**Keywords:** hepatocellular carcinoma, astaxanthin, apoptosis

## Abstract

Hepatocellular carcinoma (HCC) is a malignant tumor that can cause systemic invasion; however, the exact etiology and molecular mechanism are unknown. Astaxanthin (ASX), a powerful antioxidant, has efficient anti-oxidant, anti-inflammatory, and other activities, and has great research prospects in cancer therapy. We selected the human hepatoma cell lines, LM3 and SMMC-7721, to study the anti-tumor effect and related mechanisms of ASX. The cell lines were treated with different concentrations of ASX, and its solvent DMSO as a control, for different time periods and the results were determined using CCK8, qRT-PCR, WB, apoptotic staining, and flow cytometry. ASX induced significant apoptosis of HCC cells, and its effect may have been caused by NF-κB p65 and Wnt/β-catenin down-regulation via negative activation of PI3K/Akt and ERK. Antitumor research on ASX has provided us with a potential therapy for patients with hepatomas.

## 1. Introduction

Hepatocellular carcinoma (HCC) is a malignant tumor of the digestive system and has a high mortality rate worldwide. Jemal and colleagues reported that the incidence of HCC has continuously increased in recent years and is the fifth most common cancer and the third leading cause of cancer death [1]. HCC is a complex disease due to its polygenic, multifactorial, multi-stage evolution, and insidious onset, it is difficult to detect early, metastasizes easily and is insensitive to chemotherapy [2,3]. At present, the preferred strategy for patients diagnosed with HCC is surgical resection. According to the Barcelona Clinic Liver Cancer (BCLC) diagnostic and treatment strategy, the main curative treatment has a small range of benefits as it is aimed at asymptomatic patients in the early stage instead of those with vascular invasion and distant metastasis [4]. For those reasons, researchers have shifted their attention to molecular targets as well as transcatheter arterial chemoembolization (TACE) which have been shown to improve the survival rate of patients with HCC. However, the effects of these treatments are still not ideal due to its high recurrence rate [5,6,7,8]. Therefore, there is an urgent need to examine the molecular mechanisms of HCC development and explore potential drugs in order to increase survival rate.

The development of HCC is a multi-stage process and includes DNA repair, activation of oncogenes, inactivation of tumor suppressor genes, neovascularization, uncontrolled apoptosis, and proliferation of hepatic cells [9,10,11]. Signal transduction which induces HCC is a complex protein network with multi-channel crosstalk. Previous studies confirmed that many signal conduction pathways, such as the nuclear factor-κB (NF-κB), Wnt/β-catenin, JAK/STAT, Hedgehog, Ras/MAPK and Notch signaling pathways, showed functional disorder in HCC [12,13]. When a target is suppressed in one signal system, the tumor cells can activate downstream molecules through interactions with other pathways, thereby resulting in multidrug resistance. NF-κB, an essential nuclear transcription factor, participates in the information transfer process involving tissue damage and stress, cell differentiation, apoptosis, and tumor suppression. The effector together with the Wnt/β-catenin pathway in hepatocarcinogenesis is governed by PI3K/Akt or MAPK/ERK regulation which plays an important role in the caspase-mediated cascades and the correlation between mitochondrial apoptosis and the Bcl-2 family [14]. Fan Wang and colleagues demonstrated that salinomycin inhibits proliferation and induces apoptosis of HCC cells *in vitro* and *in vivo* through potential inhibition of Wnt/β-catenin signaling [15,16]. Verification of the role of other related drugs is urgently needed.

The anti-cancer role of antioxidants has been attracting attention, with research focused on energy metabolism and oxidative stress in cancer research. Astaxanthin (3,3′-dihydroxy-β,β′-carotene-4,4′-dione, ASX), a lipophilic compound extracted from Phaffia yeast, Haematococcus, or by chemical synthesis, has shown strong biological activities including antioxidant effects, anti-lipid peroxidation activity, anti-inflammation, cardiovascular disease prevention, and immune-modulation effects compared with other carotenoids [17,18,19]. Research by our team and previous studies have proven that ASX at higher doses is non-toxic to mice and human endothelial cells [18,19,20,21]. Related clinical studies have also been conducted into cardiovascular disease to assess the dosing, bioavailability, and safety of ASX [22]. To date, no significant side effects related to ASX have been reported [23]. Therefore, this powerful antioxidant may be a novel and potential drug for inhibiting the proliferation of carcinoma cells [17,24,25]. ASX may play an efficient role against cancer by enhancing the immune response in mice, as described by Jyonouchi and colleagues in 2000 [26]. Kowshik and other researchers found that ASX induced intrinsic apoptosis not only in oral cancer cells, but in skin cancer, breast cancer, and neuroblastoma SH-SY5Y cells [14,27,28,29,30]. Digestive tumors can be fatal, and research focused on colon cancer showed that ASX could inhibit tumor invasion by regulating the expression of ERK-2, NF-κB, and COX-2 [31]. In 2010, Tripathi DN explored the effects of ASX on early hepatocarcinogenesis in rats [32]. In addition, Song and colleagues demonstrated that ASX induced mitochondria-mediated apoptosis in rat hepatocellular carcinoma CBRH-7919 cells with an IC50 of 39 μM through inhibition of the JAK/STAT3 signaling pathway [33,34]. In human HCC cells, the protective effect of ASX has been rarely reported. Therefore, the successful application of ASX in animal models requires a better understanding of its potential protective effects in human HCC and the corresponding molecular mechanisms which may result in the development of ASX for HCC patients.

The present study was designed to evaluate the effects of ASX on the proliferation and apoptosis of HCC cells through inhibition of the transcription factors, NF-κB and β-catenin, via inactivation of the PI3K/Akt and MAPK/ERK signaling pathways using the cell counting kit (CCK8), flow cytometry, western blotting, and quantitative real-time polymerase chain reaction (qRT-PCR).

## 2. Results and Discussion

### 2.1. ASX Inhibited HCC Cell Proliferation

**Figure 1:**
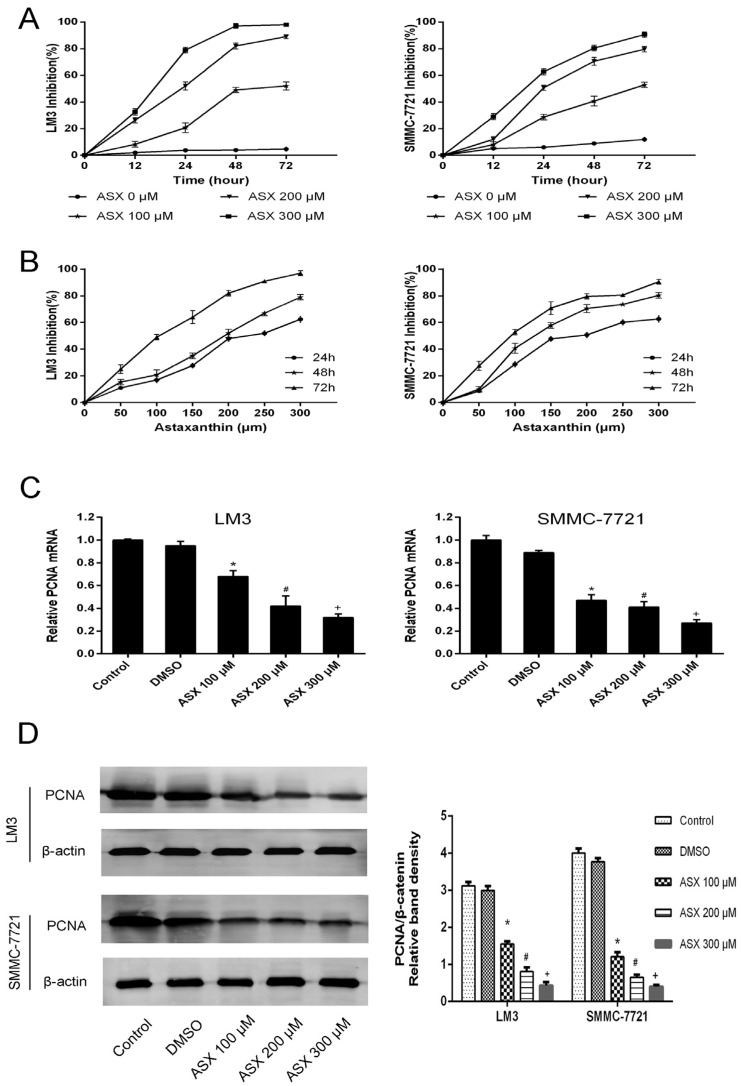
Effects of ASX on HCC cell proliferation. (**A**) LM3 and SMMC-7721 cells were treated with DMSO and ASX (100 μM, 200 μM, 300 μM) for 72 h. The effect of different concentrations of ASX on growth was lower than of DMSO and was dose-dependent; (**B**) LM3 and SMMC-7721 cells were treated with ASX (50−300 μM) and evaluated at 24 h, 48 h, and 72 h. The data showed that ASX reduced HCC cell proliferation in a time-dependent manner; (**C**) The mRNA levels of PCNA were determined by RT-PCR (*n* = 3, *^,#,+^ *p* < 0.05 for ASX *versus* DMSO); (**D**) The protein levels of PCNA were assessed by western blotting and the relative band intensities of PCNA were calculated using the Odyssey two-color infrared laser imaging system (*n* = 3, *^,#,+^ *p* < 0.05 for ASX *versus* DMSO).

Cell proliferation was determined using the CCK8 kit and the expression of proliferating cell nuclear antigen (PCNA). The HCC cell lines, LM3 and SMMC-7721, were treated with DMSO and ASX (50 μM, 100 μM, 150 μM, 200 μM, 250 μM, 300 μM), respectively, for 12 h, 24 h, 48 h, and 72 h. A cell growth curve was constructed according to the optical densities (Figure 1A,B). The data showed that ASX inhibited the growth of cancer cells in a dose- and time-dependent manner. We extracted RNA and protein from the collected cells treated with DMSO and ASX (100 μM, 200 μM, 300 μM) for 48 h and measured the gene and protein levels. The results showed that ASX reduced the expression of PCNA (Figure 1C).

### 2.2. ASX Induced Apoptosis in HCC Cells

**Figure 2:**
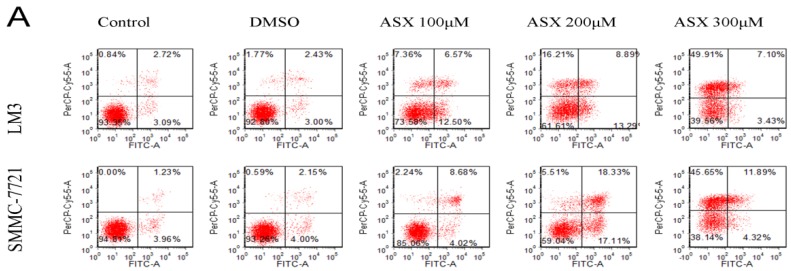

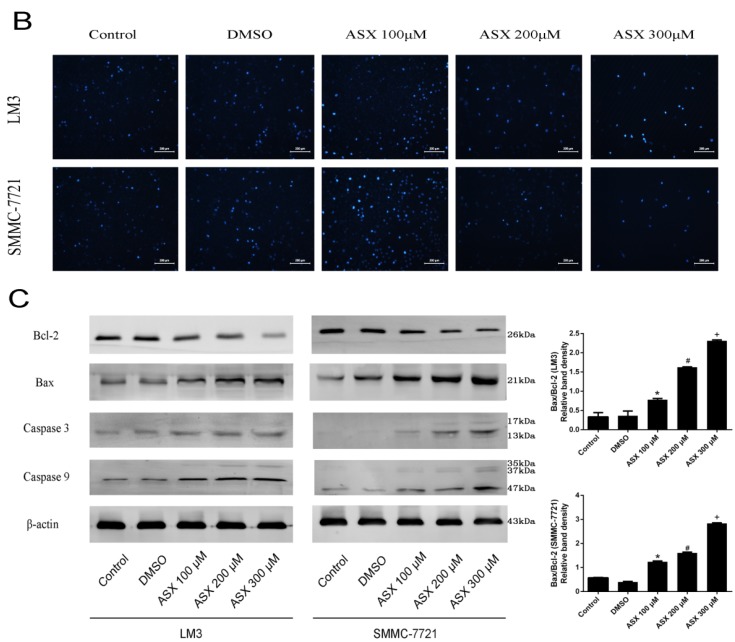
Effects of ASX on HCC cell apoptosis. (**A**) LM3 and SMMC-7721 cells were treated with DMSO and ASX (100 μM, 200 μM, 300 μM) for 48 h. Apoptosis of LM3 and SMMC-71 cells was determined by flow cytometry; (**B**) Nuclear fragmentation of LM3 and SMMC-7721 cells was observed by fluorescence microscopy after treatment with DMSO and ASX for 48 h. Magnification 200×; (**C**) The protein levels of Bcl-2, Bax, Caspase-3, and Caspase-9 were determined by western blotting. The relative band intensities of Bcl-2 and Bax were calculated using the Odyssey two-color infrared laser imaging system (*n* = 4, *^,#,+^ *p* < 0.05 for ASX *versus* DMSO).

In order to determine whether ASX induced apoptosis in LM3 and SMMC-7721 cells, we used flow cytometry, Hoechst 33342 staining and western blotting. The results showed that after ASX treatment (100 μM, 200 μM, 300 μM) for 48 h, the percentage of early and late apoptosis in HCC cells was significantly higher than that in the control and DMSO-treated cells (Figure 2A). The changed DNA located in apoptotic cells were easily combined with Hoechst 33342 reagent and showed bright blue fluorescence. Figure 2B shows the increased fluorescence intensity in ASX-treated cells which was dose-dependent. In addition, as markers of the intrinsic apoptosis pathway, the protein expression of Bcl-2, Bax, Caspase-3, and Caspase-9 following cell apoptosis was measured by western blotting and the ratio of Bax/Bcl-2 was calculated. The results were consistent with the change in number and morphology of apoptotic cells shown by flow cytometry and apoptotic staining (Figure 2C).

### 2.3. ASX Suppressed Cell Proliferation and Apoptosis by Blocking the Nuclear Translocation of NF-ΚB P65

**Figure 3:**
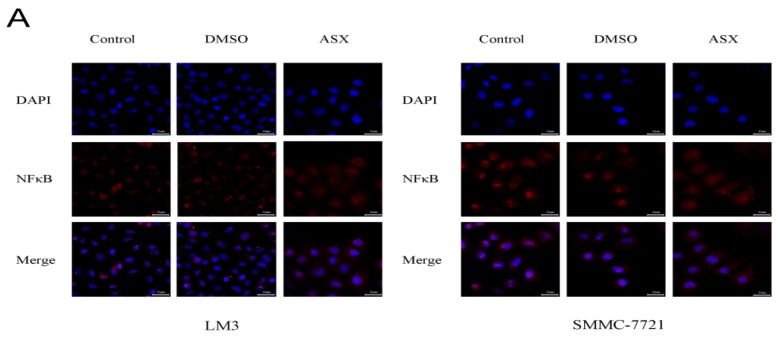

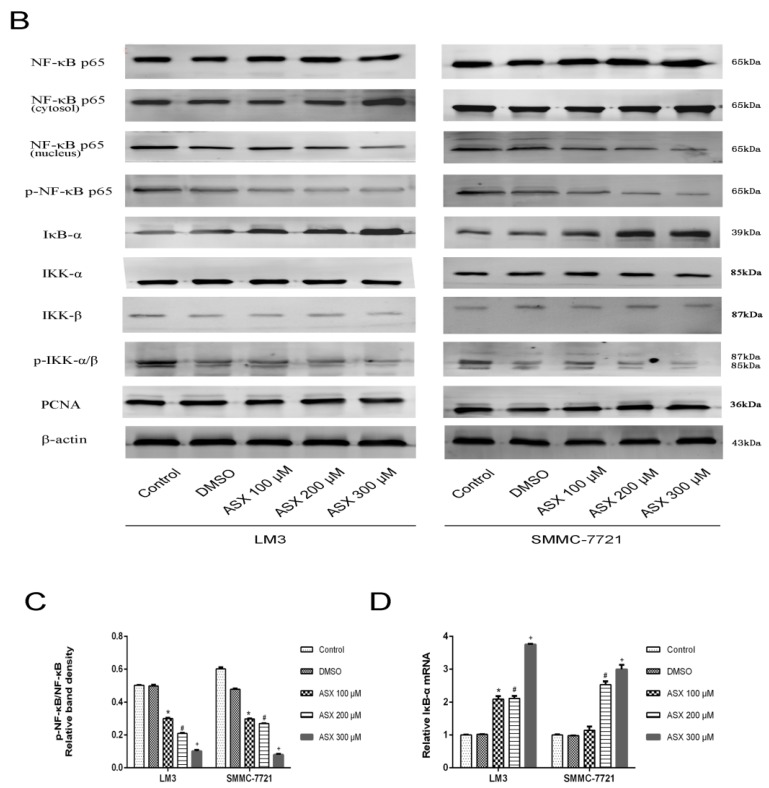
Effects of ASX on the NF-κB p65 signaling pathway. (**A**) The nuclear expression of NF-κB p65 was assessed by immunofluorescence after ASX treatment. The red fluorescence showed that NF-κB p65 and the nuclear region was dyed blue by 4′,6-diamidino-2-phenylindole (DAPI). Magnification 630×; (**B**) The protein levels of IKK-α, IKK-β, p-IKKα/β, IκB-α, NF-κB p65 and p-NF-κB p65 were determined by western blotting; (**C**) The ratio of p-NF-κB p65 and NF-κB p65 were calculated using the Odyssey two-color infrared laser imaging system (*n* = 3, *^,#,+^ *p* < 0.05 for ASX *versus* DMSO); (**D**) IκB-α mRNA was detected by RT-PCR (*n* = 3, *^,#,+^ *p* < 0.05 for ASX *versus* DMSO).

As NF-κB p65 is one of the most important pathways in inflammation and tumors, we first analyzed its nuclear translocation after treatment with ASX for 48 h. Immune confocal detection and western blotting were used to measure the expression of NF-κB p65 in the cell nucleus. As shown in Figure 3A, the red fluorescence demonstrated that NF-κB p65 in the nuclear region of ASX-treated cells was reduced in both LM3 and SMMC-7721 cells compared with the control and DMSO treatment. We then extracted the total protein, the nuclear protein, and the plasma protein which were used for the detection of NF-κB p65, respectively. The results showed that plasma protein increased, but the nuclear protein decreased on the basis of consistent total protein. The phosphorylated active NF-κB p65 showed the same results (Figure 3B,C). In addition, IκB-α, an inhibitor of NF-κB p65 signaling was used to measure its activity. As shown in Figure 3B,D, the mRNA and protein levels of IκB-α increased after ASX treatment which was consistent with our previous results.

### 2.4. ASX Suppressed Wnt/β-Catenin Signaling by Blocking the Expression and Phosphorylation of GSK-3β

**Figure 4:**
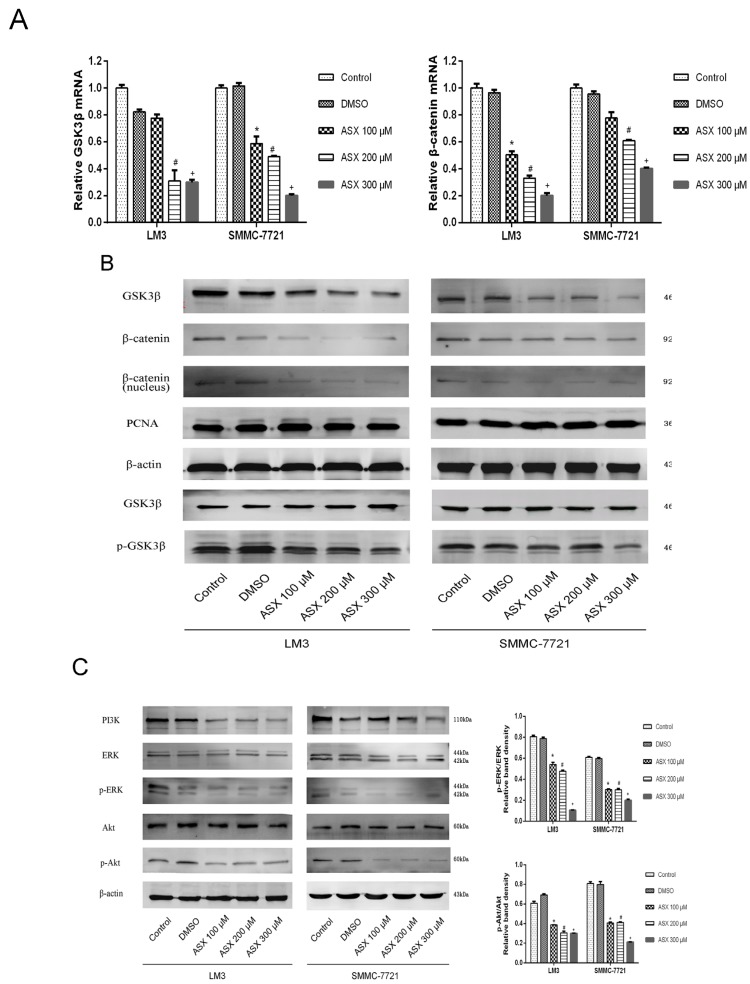

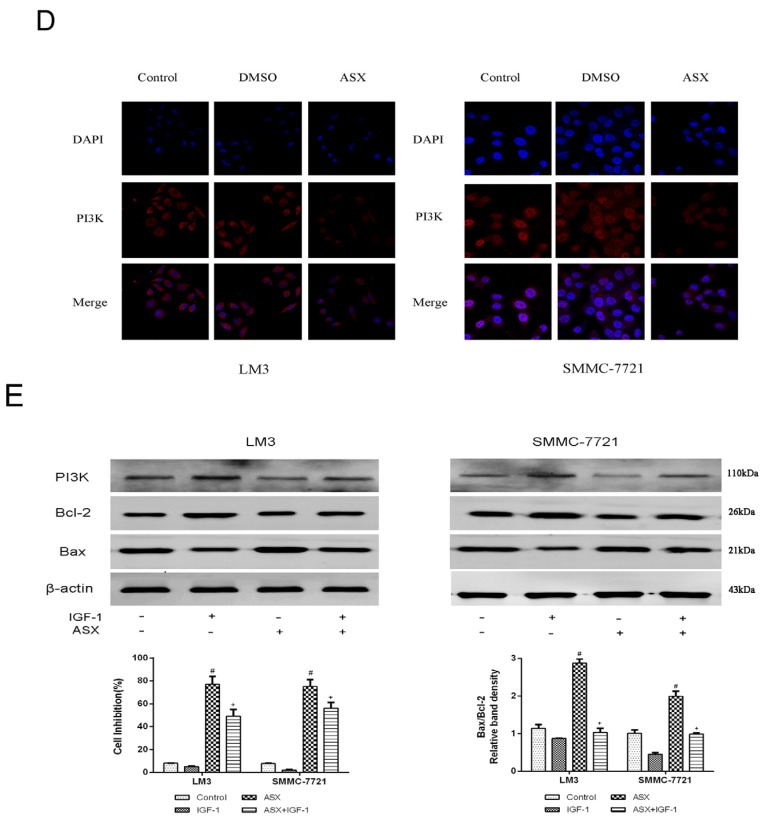
Effects of ASX on the Wnt/β-catenin signaling pathway via the reduction of GSK-3β inactivation. (**A**) The mRNA expression of GSK-3β and β-catenin was determined using real-time PCR (*n* = 3, *^,#,+^ *p* < 0.05 for ASX versus DMSO); (**B**) The protein levels of GSK-3β, β-catenin and p-GSK-3β were determined by western blotting; (**C**) The protein levels of PI3K, ERK, p-ERK, Akt, and p-Akt were determined by western blotting. The ratio of p-ERK and ERK, p-Akt, and Akt were calculated using the Odyssey two-color infrared laser imaging system (*n* = 4, *^,#,+^ *p* < 0.05 for ASX *versus* DMSO); (**D**) The expression of PI3K was assessed by immunofluorescence after ASX treatment. The red fluorescence showed that PI3K and the nuclear region was dyed blue by 4′,6-diamidino-2-phenylindole (DAPI). Magnification 630×; (**E**) The protein levels of PI3K, Bax, Bcl-2, and β-actin in LM3 and SMMC-7721 were determined by western blotting. The relative band intensities of Bcl-2 and Bax were calculated using the Odyssey two-color infrared laser imaging system. The inhibition rates were evaluated by CCK8 (*n* = 4, ^#^*p* < 0.05 for ASX *versus* Control, ^+^ *p* < 0.05 for ASX + IGF-1 *versus* ASX).

The Wnt signaling pathway, which has been shown to play an important role in the formation of liver cancer, has crosstalk with NF-κB p65. During this crosstalk, the expression of GSK-3β and its phosphate levels had effects on regulation of Wnt and NF-κB p65 pathways. We determined the gene expression of GSK-3β and β-catenin in HCC cells using qRT-PCR. The mRNA levels in LM3 and SMMC-7721 cells decreased in a dose-dependent manner (Figure 4A). The protein expression of GSK-3β and β-catenin as shown by western blotting was reduced in a dose-dependent manner. We measured the phosphorylation of GSK-3β after ensuring consistent expression in the different groups. The inactive form of GSK-3β was reduced consistently with the suppressed nuclear transfer of β-catenin (Figure 4B). Based on the inhibition of NF-κB and Wnt/β-catenin signals by ASX, we further explored its upstream pathways. As shown in Figure 4C, the protein levels were reduced following ASX treatment, accompanied by a decline in the phosphorylation of Akt and ERK. The activator of PI3K used in the ASX treatment groups resulted in a reduced ratio of Bax to Bcl-2 with a lower cell inhibition rate. In summary, PI3K, Akt, and ERK were negatively regulated by ASX and inhibition of the NF-κB p65 and Wnt/β-catenin pathways may be restricted by the inactivation of their upstream kinases, PI3K/Akt, and ERK, as a means of regulation.

## 3. Discussion

The development of HCC, which is a serious threat to human health, involves complex mechanisms. Conventional curative treatments including mainly surgery and interventional treatment are only appropriate in 30%–40% of patients with early stage liver cancer [2,35,36]. Therefore, researchers have focused on effective drugs, such as alkylating agents, antimetabolites, antibiotics, and hormones. Emerging data have indicated that antioxidant drugs also play an important role in cancer therapy [37,38,39]. The carotenoid, ASX, is 10 times greater than zeaxanthin, lutein, and canthaxanthin, and 100 times greater than α-tocopherol in eliminating oxygen free radicals, and has attracted the attention of scientists [17]. Studies on the human hepatoma cell lines, LM3 and SMMC-7721, showed that ASX induced tumor cell apoptosis and inhibited proliferation which was related to the NF-κB p65 and Wnt/β-catenin pathways.

The proliferation and differentiation of tumor cells are involved in tumor invasion, therefore, inhibition of growth and promotion of apoptosis in HCC cells are important targets in cancer treatment [2,5]. In our study, we chose CCK8, flow cytometry, Hoechst 33342 staining, qRT-PCR and western blotting to comprehensively study the proliferation and apoptosis of the HCC cell lines LM3 and SMMC-7721. To assess proliferation, we measured the growth of HCC cells within 72 h treated with different concentrations of ASX (50–300 μM) using the CCK8 kit. The results showed that ASX inhibited tumor cell proliferation in a time- and dose-dependent manner. In cells treated with high dose ASX, strong inhibition was seen at 72 h. We selected three effective concentrations for our experiments. PCNA, an index used in cell proliferation studies, is associated with the synthesis of cell DNA, and reflects the state of cell proliferation [40]. The gene and protein levels of PCNA showed that the rate and activity of tumor cell proliferation was reduced by ASX (100 μM, 200 μM, and 300 μM) treatment. Apoptosis resistance has also been found to be the main cause of tumorigenesis and drug resistance [41]. We evaluated the number and morphology of apoptotic cells using flow cytometry and Hoechst staining. An increase in apoptotic cells and their obvious morphological changes were consistent with the decline in anti-apoptotic Bcl-2 and the increase in pro-apoptotic Bax following ASX treatment [42]. These findings showed that the proliferative ability of HCC cells decreased, and the incidence of apoptosis increased after treatment with 100 μM, 200 μM, and 300 μM ASX. We showed that ASX inhibited the invasion of tumor cells via NF-κB p65 and Wnt/β-catenin by reducing the phosphorylation of GSK-3β (Ser9) in liver oncogenesis [14,26,43,44].

The occurrence of liver cancer is a multifactorial heterogeneous process characterized by key hallmarks which occur via the aberrant activation of transcription factors such as NF-κB p65 and β-catenin [45,46,47]. NF-κB, a pro-survival transcription factor is widely found in various tissues and organs, and can promote tumorigenesis, tumor cell proliferation, invasion, and metastasis [46]. Qiao and colleagues demonstrated that adenovirus-mediated transfer of mutant IκBα potently inhibited NF-κB activity, and this enhanced oxidative stress-induced cell killing in the hepatoma cells line Huh7, was also found in other cancer cells [48]. The above experimental results showed decreased NF-κB p65 and increased IκBα at the gene and protein level in both hepatoma cell lines, indicating that ASX can down-regulate the NF-κB p65 pathway. ASX increased the ratios of ADP/ATP and GDP/GTP to inactivate the Ras signal. This negative regulation inhibited the phosphorylation of PI3K and subsequently prevented the second messenger, PIP3, activating Akt, a Ser/Thr protein kinase. Thus, the 23rd threonine of IκKα was not phosphorylated and did not inhibit the nuclear localization signals of NF-κB, as shown by the expression level of PI3K/Akt and ERK. The anti-apoptotic Bcl-2 gene with a κB site which did not combine with NF-κB showed lower expression, while the pro-apoptotic Bax had no κB sites, thereby, the Bax/Bcl-2 ratio changes had mitochondrial apoptotic roles [42,49]. In addition, reduced nuclear transcription attenuated caspase-mediated apoptosis through the IAP, JNK, and FLIP pathways which promoted the killing of tumor cells [43].

**Figure 5:**
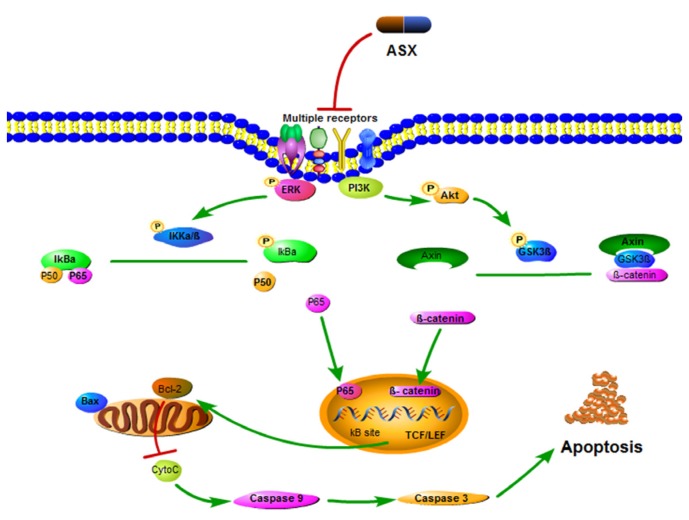
Mechanism of ASX action. ASX made the conformational change of PI3K and inhibited the phosphorylation of Akt and ERK which induced the Wnt protein to combine with the receptors, and thereby reduced the phosphorylation of IKKα/β (Ser176/180) and GSK-3β (Ser9). The reduced β-catenin and p-NFκB inhibited Bcl-2 transcription, which changed the Bax/Bcl-2 ratio. The caspases associated with mitochondrial apoptosis were then activated to induce cell death.

Abrogation of NF-κB signaling by ASX was associated with coordinated inhibition of Wnt/β-catenin signaling which is involved in cellular proliferation and apoptosis regulated by the PI3K/Akt and ERK signaling pathways [50,51]. In cells, β-catenin can combine with Axin, APC and GSK-3β to form degradation complexes. When glycogen synthase kinase 3β (GSK-3β), a serine/threonine kinase of the canonical Wnt/β-catenin pathway, is phosphorylated to the inactive form, β-catenin is released to start transcription [52]. In normal mature cells, inactive Wnt/β-catenin can initiate transcription of downstream target genes once activated by abnormal factors, leading to the occurrence of cancers. The described conformational change in PI3K inhibited phosphorylation of Akt and ERK which induced the Wnt protein to combine with the receptors of frizzled transmembrane proteins located at the cell surface and low-density lipoprotein, and thereby reduced the phosphorylation of GSK-3β (Ser9) [53]. Thus, non-catalyzed β-catenin showed a polymer form, but was unable to be transferred to the nucleus [44,54]. Finally, the isolated T cell factor (TCF/LEF) similarly changed the expression of target genes, such as Bcl-2 and Caspase-3 to control cell proliferation and malignant transformation (Figure 5). Our experimental results showed that the expression of the above proteins changed signal transductions in LM3 and SMMC-7721 cells.

In this study, we demonstrated that ASX inhibited proliferation and induced apoptosis of human HCC cells *in vitro* via the NF-κB and Wnt/β-catenin signaling pathways. ASX attenuated cell proliferation as shown by CCK8 and the expression of PCNA protein. The results of flow cytometry and the Bax/Bcl-2 ratio showed that ASX had anti-apoptotic effects via the NF-κB p65 and Wnt/β-catenin pathways and inhibited PI3K/Akt and ERK as shown by western blotting and qRT-PCR. Our findings demonstrate that ASX is a promising potential therapeutic agent for human HCC, and has antioxidant, anti-atherosclerosis and anti-inflammatory activity.

## 4. Experimental Section

### 4.1. Cell Lines and Culture

The HCC cell lines, LM3 and SMMC-7721, were purchased from the Cell Bank of the Chinese Academy of Sciences Committee Type Culture Collection (Shanghai, China). The two cell lines were cultured in high glucose Dulbecco’s modified Eagle’s medium (DMEM, Thermo, Shanghai, China) with 10% fetal bovine serum (Hyclone, Logan, UT, South America), 100 U/mL penicillin, and 100 μg/mL streptomycin (Gibco, Burlington, Canada) in a 5% CO_2_ and 95% air incubator at 37 °C. The cells were sub-cultured when the cell density reached 90%.

### 4.2. Chemicals

ASX and insulin-like growth factor 1 (IGF-1) were purchased from Sigma Aldrich (St. Louis, MO, USA) and dissolved in dimethyl sulfoxide (DMSO, Sigma, St. Louis, MO, USA) to yield a 20 mM stock solution and stored at −20 °C for future use. The cell counting kit (CCK8) was produced by Dojindo (Dojindo Laboratories, Tokyo, Japan). The ribonucleic acid (RNA) polymerase chain reaction (PCR) kit was purchased from Takara (Takara Biotechnology, Dalian, China). Antibodies for PCNA, Bcl-2, Bax, Caspase-3, Caspase-9, NF-κB p65, p-NF-κB p65 (Ser276), IκB-α, β-catenin, PI3K, Akt, p-Akt (Ser473), ERK, p-ERK (Thr202/Tyr204), GSK-3β, p-GSK-3β (Ser9), IKKα, IKKβ, p-IKKα/β (Ser176/180) and β-actin were purchased from Cell Signaling Technology (Danvers, MA, USA). The Annexin V-APC/7-ADD apoptosis detection kit was purchased from BD Biosciences (San Jose, CA, USA).

### 4.3. Cell Proliferation and Viability

The HCC cell lines, LM3 and SMMC-7721, were plated at a density of 2 × 10^4^ cells/mL in 96-well plates (100 μL medium per well). One day after seeding, the HCC cells were treated with ASX [20,30,33,34] and five replicates were included for each concentration. Cell proliferation and viability were measured using the CCK8 and a microplate reader (Synergy H4, BioTek, Winooski, VT, USA) at a wavelength of 450 nm.

### 4.4. Cell Apoptosis Analyses Using Flow Cytometry

Hepatoma cells in logarithmic growth phase were seeded into six-well plates at a density of 1 × 10^6^ cells/mL and exposed to ASX for 48 h. The processed cells were respectively added to Falcon tubes at a density of 1 × 10^6^ cells/mL containing 100 μL of 1× binding buffer. These cells were incubated for 20 min at room temperature with annexin-V/APC supplemented with 7-AAD (BD Biosciences).

### 4.5. Hoechst 33342 Staining

Fixed cultured cells were collected after ASX treatment at a density of 1 × 10^6^ cells/mL. After 48 h, they were washed with PBS and mixed with Hoechst 33342 stain (1 μL added to 200 μL PBS) solution (Sigma Aldrich). The filled 12-well plates were placed at 4 °C in the dark for 20 min. Fluorescence microscopy (Leica, Wetzlar, Germany) was used to examine the blue fluorescent cells. 4.6. Reverse Transcription-Polymerase Chain Reaction and qRT PCR

Total RNA was extracted and then transcribed into cDNA using the reverse transcription kit (TaKaRa Biotechnology, Dalian, China). According to the manufacturer’s protocol, a 7900HT fast real-time PCR system (Applied Biosystems, foster, CA, USA) was used to determine the gene expression level of PCNA, IκB-α, β-catenin, PI3K, and GSK-3β. Primers used in the experiment are shown in Table 1.

**Table 1:** Nucleotide sequences of primers used for qRT-PCR.

Gene		Primer Sequence (5′-3′)
*PCNA*	Forward	GCTGACATCGGACACTTA
	Reverse	CTCAGGTACAAACTTGGTG
*IκB-α*	Forward	TGAAGGACGAGGAGTACGAGC
	Reverse	TGCAGGAACGAGTCTCCGT
*β-catenin*	Forward	TACCGTTGGATTGATTCG
	Reverse	GTCAGAGGTGCTGTGGCT
*PI3K*	Forward	CCACGACCATCATCAGGTGAA
	Reverse	CCTCACGGAGGCATTCTAAAGT
*GSK-3β*	Forward	AGACGCTCCCTGTGATTTATGT
	Reverse	CCGATGGCAGATTCCAAAGG
*β-actin*	Forward	CTGGAACGGTGAAGGTGACA
	Reverse	AAGGGACTTCCTGTAACAATGCA

### 4.6. Western Blot Analysis

Nuclear and cytoplasmic extracts were prepared as described using a nucleoprotein and cytoplasm protein extraction kit (Keygen, Nanjing, China). The cells were collected after being washed with precooled PBS and 200 μL Buffer A was added. Oscillation for 15 s was then carried out and the cells were placed in an ice-bath for 15 min. Buffer B (11 μL) was added and the cells were oscillated for 5 s before being placed in an ice-bath for 1 min. Following centrifugation at 16,000× *g* for 5 min at 4 °C, the supernatant was obtained containing the cytoplasm protein. Buffer C (100 μL) was added to the centrifugal sediment, oscillated for 15 s and then placed in an ice-bath for 40 min. The nuclear protein was collected following centrifugation at 16,000× *g* for 10 min. The bicinchoninic acid (BCA) protein assay (Thermo Scientific) was used to determine the concentration of the prepared protein which was then mixed with 5× sodium dodecyl sulfate-polyacrylamide gel electrophoresis (SDS-PAGE) sample loading buffer. Equivalent amounts of protein were boiled and subjected to SDS-polyacrylamide gels and transferred onto polyvinylidene fluoride (PVDF) membranes which were blocked for 60 min with 5% bovine serum albumin (BSA) dissolved in PBS. The blots were then incubated overnight at 4 °C with the following antibody concentrations: PCNA (1:500), Bcl-2 (1:500), Bax (1:1000), Caspase-3 (1:500), Caspase-9 (1:500), NF-κB p65 (1:500), p-NF-κB p65 (Ser276) (1:200), IκB-α (1:500), β-catenin (1:500), PI3K (1:1000), Akt (1:1000), p-Akt (Ser473) (1:500), ERK (1:1000), p-ERK (Thr202/Tyr204) (1:500), GSK-3β (1:1000), p-GSK-3β (Ser9) (1:500), IKKα (1:500), IKKβ (1:500), p-IKKα/β (Ser176/180) (1:500), and β-actin (1:1000). PBS containing 0.1% Tween 20 (PBST) was used to wash the membranes three times before and after incubation with the secondary antibody (anti-rabbit or anti-mouse IgG (1:2000)) for 1 h at room temperature.

### 4.7. Statistical Analyses

The experimental data were evaluated by calculating the mean ± SD analyzed by SPSS 20.0 software (IBM, Armonk, NY, USA). Student’s t test and one-way analysis of variance (ANOVA) were performed to compare the results of the CCK8 assay and real-time PCR. The bar charts were obtained using GraphPad Prism Software version 6.0 for Windows (GraphPad, San Diego, CA, USA). *p* < 0.05 was considered statistically significant.

## 5. Conclusions

Astaxanthin induced significant apoptosis of HCC cells, and its effect may have been caused by NF-κB p65 and Wnt/β-catenin down-regulation via negative activation of PI3K/Akt and ERK. Antitumor research on ASX has provided us with a potential therapy for patients with hepatomas.

## Abbreviations

ASX: astaxanthin
HCC: hepatocellular carcinoma
NF-κB: nuclear factor-κB
JAK: janus kinase
STAT: signal transducers and activators of transcription
MAPK: mitogen-activated protein kinase
PI3K: phosphatidylinositol 3-kinase
ERK: extracellular signal regulated kinases
PCNA: proliferating cell nuclear antigen
GSK-3β: glycogen synthase kinase 3β
